# Ginseng^®^ Alleviates Malathion-Induced Hepatorenal Injury through Modulation of the Biochemical, Antioxidant, Anti-Apoptotic, and Anti-Inflammatory Markers in Male Rats

**DOI:** 10.3390/life12050771

**Published:** 2022-05-23

**Authors:** Heba I. Ghamry, Asmaa A. Aboushouk, Mohamed Mohamed Soliman, Sarah M. Albogami, Hossam G. Tohamy, Osama S. El Okle, Saed A. Althobaiti, Shaymaa Rezk, Foad Farrag, Azza I. Helal, Hanan A. Ghoneim, Mustafa Shukry

**Affiliations:** 1 Department of Home Economics, College of Home Economics, King Khalid University, P.O. Box 960, Abha 61421, Saudi Arabia; hgmry@kku.edu.sa; 2 Department of Pathology, Faculty of Veterinary Medicine, Alexandria University, Alexandria 22758, Egypt; asmaaali_199217@yahoo.com (A.A.A.); hossam.gafaar@alexu.edu.eg (H.G.T.); 3 Clinical Laboratory Sciences Department, Turabah University College, Taif University, Taif 21995, Saudi Arabia; 4 Department of Biotechnology, College of Science, Taif University, P.O. Box 11099, Taif 21944, Saudi Arabia; dr.sarah@tu.edu.sa; 5 Department of Forensic Medicine and Toxicology, Faculty of Veterinary Medicine, Alexandria University, Alexandria 22758, Egypt; osama.forensics@alexu.edu.eg; 6 Biology Department, Turabah University College, Taif University, Taif 21995, Saudi Arabia; saed@tu.edu.sa; 7 Department of Histology, Faculty of Veterinary Medicine, Mansoura University, Mansoura 35516, Egypt; rezk_2010@yahoo.com; 8 Department of Anatomy and Embryology, Faculty of Veterinary Medicine, Kafrelsheikh University, Kafrelsheikh 33516, Egypt; foad.farrag@vet.kfs.edu.eg; 9 Department of Histology and Cell Biology, Faculty of Medicine, Kafrelsheikh University, Kafrelsheikh 33516, Egypt; helal_2010@yahoo.com; 10 Department of Physiology, Faculty of Veterinary Medicine, Damanhour University, Damanhour 22516, Egypt; ghoneim_2000@yahoo.com; 11 Department of Physiology, Faculty of Veterinary Medicine, Kafrelsheikh University, Kafrelsheikh 33516, Egypt

**Keywords:** malathion, hepato-renal injury, Ginseng^®^, antioxidant, HO-1, 8-OHdG

## Abstract

This study aims to see if Ginseng^®^ can reduce the hepatorenal damage caused by malathion. Four groups of forty male Wistar albino rats were alienated. Group 1 was a control group that got orally supplied corn oil (vehicle). Group 2 was intoxicated by malathion dissolved in corn oil orally at 135 mg/kg/day. Group 3 orally received both malathion + Panax Ginseng^®^ (300 mg/kg/day). Group 4 was orally given Panax Ginseng^®^ at a 300 mg/kg/day dose. Treatments were administered daily and continued for up to 30 consecutive days. Malathion’s toxic effect on both hepatic and renal tissues was revealed by a considerable loss in body weight and biochemically by a marked increase in liver enzymes, LDH, ACP, cholesterol, and functional renal markers with a marked decrease in serum TP, albumin, and TG levels with decreased AchE and Paraoxonase activity. Additionally, malondialdehydes, nitric oxide (nitrite), 8-hydroxy-2-deoxyguanosine, and TNFα with a significant drop in the antioxidant activities were reported in the malathion group. Malathion upregulated the inflammatory cytokines and apoptotic genes, while Nrf2, Bcl2, and HO-1 were downregulated. Ginseng^®^ and malathion co-treatment reduced malathion’s harmful effects by restoring metabolic indicators, enhancing antioxidant pursuit, lowering the inflammatory reaction, and alleviating pathological alterations. So, Ginseng^®^ may have protective effects against hepatic and renal malathion-induced toxicity on biochemical, antioxidant, molecular, and cell levels.

## 1. Introduction

Malathion, one of the first organophosphate pesticides, is still widely used in Egypt, particularly for agriculture. When malathion has acute toxicity, the nervous system is the primary target. It is characterized by overstimulation of the cholinergic pathways. Chlonergic pathways are overstimulated due to the lack of acetylcholinesterase (AChE) and butyrylcholinesterase (BchE) enzymes. [1]. Even at low dosages, Malathion exposure frequently results in severe liver and kidney damage in laboratory animals [2,3]. 

Malathion’s widespread usage contributes to environmental contamination and raises the risk of exposure [4,5]. Excessive exposure can result in acute or chronic poisoning, particularly in underdeveloped countries [6]. Malathion is considered a moderately toxic insecticide by the World Health Organization (WHO) [7]. Prescribing a dose of 2 g/m^2^, with a residual consequence of two to three months, the world health organization’s extreme standard intake is 0.02 mg/kg/day [8]. More than 30 million pounds of malathion are used each year, according to the Environmental Protection Agency (EPA). Due to malathion’s lipophilicity, it is rapidly absorbed and dispersed throughout the body, resulting in various diseases [9]. Malathion-induced hyperglycemia can potentially be explained by its damaging inflammatory effects on the liver [10]. Excessive oxidative damage has been found in human cells exposed to malathion, increasing the creation of reactive oxygen species [11,12,13]. It alters tissue antioxidant endogenous enzymatic activities and nonenzymatic levels [14]. So, it can lead to mitochondrial malfunction, DNA breakage, and apoptosis [4]. It was found that malathion can cause liver tissue damage and hepatocellular injury [15]. 

For centuries, Panax Ginseng^®^ (Araliaceae) has been utilized in East Asia as a food supplement or herbal treatment [16]. Additionally, it can reduce inflammation, blood sugar, blood lipids, and cancer-causing free radicals [17] and prevent chronic fatigue and cardiovascular, digestive, and age-related conditions [18]. Triterpenes, saponins, essential oils, alkaloids, aminoglycosides, fatty acids, peptidoglycan, polysaccharides, vitamins, minerals, and phenolic compounds are vital components found in Ginseng^®^ extracts. There are many types of ginsenosides in Ginseng^®^ plants, and they are crucial in the plant’s physiological and pharmacological qualities [19]. The hepato-renoprotective effects of Ginseng^®^ have been highlighted in many studies [20,21] due to its anti-inflammatory, anti-apoptotic, and antioxidant attributes [21,22]. Malathion-related hepatorenal injury is the subject of a few clinical trials. Consequently, this study’s primary objective is to determine whether Ginseng^®^ can protect rats against malathion-induced hepato-renal injury. 

## 2. Materials and Methods

### 2.1. Chemicals

Panax Ginseng^®^ powder root extracts 3.5% as manufactured indicated (Ginseng^®^, 100 mg soft gelatin capsules; PHARCO pharmaceuticals, Alexandria, Egypt). Malathion (98% active ingredient, O-dimethyl phosphorodithioate of diethyl mercaptosuccinate) was found in Kafr el-zayat, Egypt. The powder was soaked in 70% aqueous ethanol for ten days at 25 °C and filtered. The solution evaporated in vacuo was semi-gelatinous. All other chemicals used were of the highest analytical grade. 

### 2.2. Experimental Animals, Treatment Design

Forty albino male Wistar Wistar rats weighed an average of (149 ± 5 g). Rats were obtained from the Medical Research Institute at Alexandria University, Egypt, and were fed regular food and allowed free access to water. They were kept under close supervision, primarily in metallic rat cages with a 12-h light-dark cycle and a temperature of 27 °C ± 2 for a 10-day adaptation period before treatment. After the acclimatization period, four experimental groups of rats were arbitrarily and similarly alienated (*n* = 10). Group 1 were provided solely malathion-carrying corn oil as a control group. Group 2 was given malathion dissolved in corn oil at 135 mg/kg/orally [23]. Group 3 was given orally both malathion + Panax Ginseng^®^ (300 mg/kg/day, orally) [24,25,26]. Group 4 was given Panax Ginseng^®^ at (300 mg/kg/day orally. Treatments were administered daily and continued for up to 30 consecutive days.

Rats were anesthetized with ketamine and xylazine injections on the end day of the trial, with recorded body weight. Serum was separated from blood drawn from the orbital venous plexus to analyze hepatic functional biomarkers using a centrifuge set to 3000 rpm for 10 min. Rats were decapitated, and the liver and kidney were isolated and weighed, then rinsed in ice-cold saline. A 10% neutral-buffered formalin solution was used to preserve tissue for histopathological analysis. To analyze oxidative stress markers, another liver slice was held at −20 °C, while the final one was kept at −80 °C for gene expression.

### 2.3. Biochemical Investigation

Serum aspartate aminotransferase (AST) and alanine aminotransferase (ALT) enzymes were assessed following the manufacture [27], and the alkaline phosphatase (ALP) according to the method [28]. Analysis of total protein and albumin was carried out [29]. Albumin was subtracted from total protein, and the serum globulin level was determined. Serum urea and creatinine levels were also assessed [30,31]. Serum uric acid was tested following [32], using commercially available kits (Spinreact, S.A., Gerona, Spain). 

Diamond Diagnostics kits, (Cairo, Egypt) and ELISA kits obtained from Wuhan EIAab Science Co. (Catalogue No; E1864r, Wuhan, China) were used to analyze acid phosphatase (ACP) and the enzyme lactate dehydrogenase (LDH) in serum samples, respectively. 

Colorimetric kits from Boehringer Mannheim were used to quantity serum triglycerides (TG) and total cholesterol (TC) (Mannheim, Germany). HDL-C was measured according to Lopes-Virella et al. [33]. A serum sample was precipitated with phosphotungstic acid and magnesium chloride, and the cholesterol concentration was measured in the clear supernatant using the Boehringer Mannheim kit (Mannheim, Germany). After that, LDL-C was calculated following the Friedewald et al. [34] equation:

$$\mathrm{LDL}-C=\mathrm{TC}-\left(\mathrm{HDL}-C+\frac{1}{5}\mathrm{TG}\right)$$

Acetylcholinesterase (AChE) was assessed using an ELISA kit, NOVA (Bioneovan Co., Ltd., DaXing Industry Zone, Beijing, China). Auto analyzers and commercial kits measured the serum’s paraoxonase (PON) activities (Rel assay, Gaziantep, Turkey). (Cobas Integra 800, Roche, Basel, Switzerland). The ammonia concentration was measured using an ammonia kit (Abcam, Cambridge, UK).

### 2.4. Antioxidant Tissue Parameters Analysis

Tumor necrosis factor-alpha was detected (EZMTNFA, Millipore, Burlington, MA, USA). Detection of lipid peroxidation (LPO) in terms of malondialdehyde (MDA) formation using spectrophotometry followed by Ohkawa et al. [35] Nitric oxide (NO) assessed calorimetrically following Green et al. [36] Glutathione (GSH) was measured according to the method of Ellman [37]. A yellow chemical spectrophotometrically measured at 405 nm after GSH reduction of 5,5′-dithiobis (2-nitrobenzoic acid) SOD and CAT were found to be measured following Sun et al. [38] and Aebi [39], correspondingly. The activity of glutathione peroxidase (GPx) was assessed following Paglia and Valentine [40].

OxiSelect^TM^ Oxidative DNA Damage ELISA Kit (Cell Biolabs, San Diego, CA, USA) was used to analyze 8-hydroxydeoxyguanosine (8-OHdG), a DNA damage marker.

### 2.5. Gene Expression

According to the manufacturer’s instructions, total RNA was extracted using the TRIzol reagent (Life Technologies, Gaithersburg, MD, USA), and cDNA was generated directly using the MultiScribe RT enzyme kit (Applied Biosystems, Foster City, CA, USA). A 7500 Real-Time PCR System (Applied Biosystems, Life Technologies, CA, USA) exposed the cDNA in triplicate for real-time PCR analysis (Applied Biosystems, Foster City, CA, USA) using SYBR Green PCR Master Mix. 

Compared to the control, the mRNA expression fold change in the genes under study was calculated. GAPDH housekeeping gene were utilized to normalize the mRNA expression of the genes that were being assessed. The primer’s sequences are listed in Table S1.

### 2.6. Histopathological Examination

Liver and kidney samples were fixed in 10% neutral-buffered formalin for at least 24 h; tissue samples were then paraffin-embedded using the standard procedure [41]. Paraffin blocks were sectioned into five-micron thick slices stained with HE and viewed under a light microscope.

### 2.7. Statistical Analysis

The data were analyzed with a one-way analysis of variance (SPSS) (version 25). *p* < 0.05 were considered significant, and data were available as means ± SEM. The significant main effects of the experimental treatment were examined using Duncan’s multiple range test.

## 3. Results

### 3.1. Bodyweight 

As shown in Table 1, malathion-intoxicated rats had significantly lower final body weights than other groups. The liver and kidney absolute and relative weights were within normal ranges. Our result also showed that the co-treatment of Ginseng^®^ and malathion reduces the adverse effects on rat growth. 

### 3.2. Liver and Kidney Serum Markers

As shown in Table 2, there were significant upsurges in the serum activities of liver enzymes such as AST, ALT, ALP, LDH, and ACP in addition to the serum levels of total cholesterol, urea, creatinine, uric acid, and ammonia in the rats of the malathion intoxicated group in contrast to the control rats. At the same time, they displayed a significant decrease in the TP, albumin, and TG serum levels. Conversely, AchE and Paraoxonase activity was downgraded. The co-treatment of malathion intoxicated rats with Ginseng^®^ showed significant decrements in the serum activities of the previously mentioned liver enzymes and the serum levels of total cholesterol, urea, creatinine, and uric acid, with substantial increments in serum TP, albumin, and TG as compared with the malathion intoxicated rats. In contrast, no significant alterations were observed in the Ginseng^®^-treated rats in all measured biochemical parameters concerning the control group.

### 3.3. Hepatic and Renal Oxidative Stress Markers

**Figure 1:**
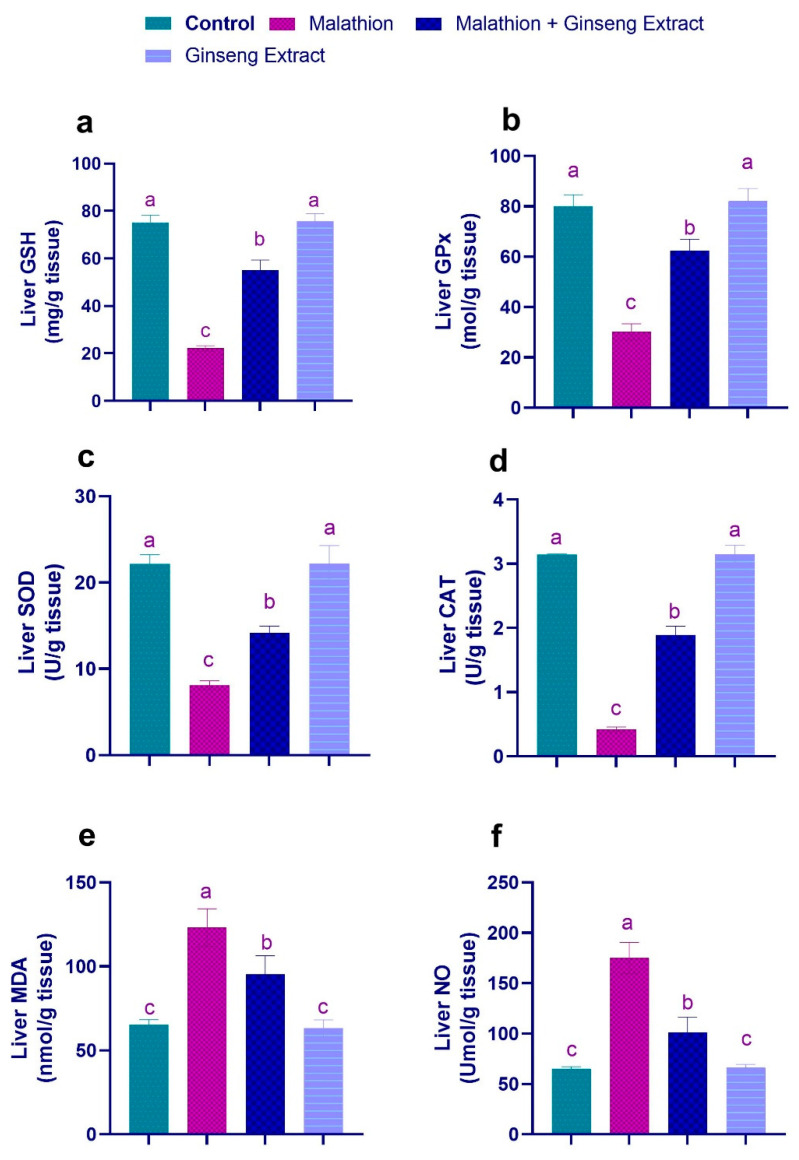
Ginseng^®^’s effect on oxidative stress caused by malathion in hepatic tissue (**a**) GSH (**b**) GPX (**c**) SOD. (**d**) CAT (**e**) MDA (**f**) NO. Data were expressed as mean ± SEM. ^a–c^. Mean values within the columns having different superscript letters are significantly different at *p* < 0.05. *n* = 7 rats.

**Figure 2:**
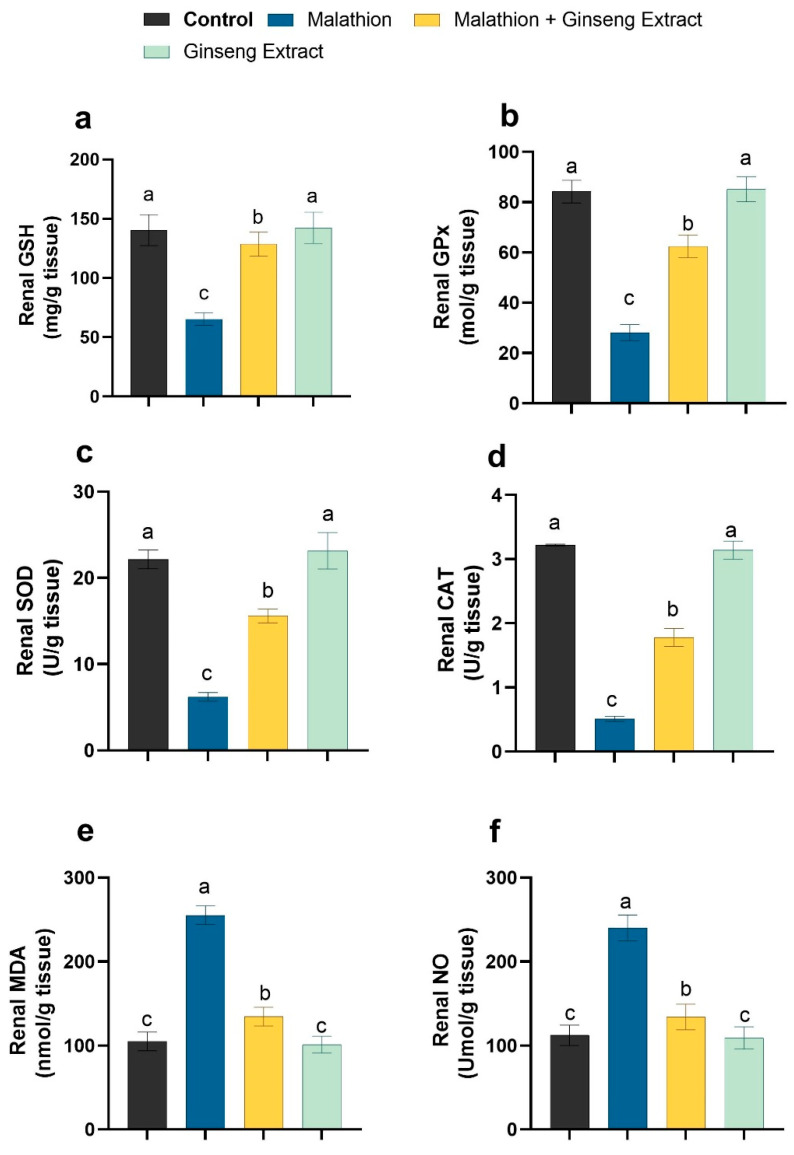
Ginseng^®^’s effect on oxidative stress caused by malathion in renal tissue (**a**) GSH (**b**) GPX (**c**) SOD. (**d**) CAT (**e**) MDA (**f**) NO. Data were presented as mean ± SEM. ^a–c^. Mean values within the columns having different superscript letters are significantly different at *p* < 0.05. *n* = 7 rats.

As shown in Figure 1 and Figure 2, compared to control rats, the malathion intoxicated group showed significant decrements in the liver and kidney tissue non-enzymatic GSH concentration and enzymatic GPX, SOD, and CAT pursuits. At the same time, it revealed significant increments in the concentration of hepatic MDA and NO as oxidative stress indicators. Conversely, the concurrent treatment of malathion intoxicated rats with Ginseng^®^ exhibited substantial increases in the hepatic and renal GSH level and hepatic activities of GPX, SOD, and CAT with a significant reduction in the concentrations of hepatic and renal MDA and NO when compared to the malathion intoxicated rats only. The antioxidant and oxidant biomarker readings of the Ginseng^®^-treated rats did not differ significantly from those of the control rats.

### 3.4. Genes Expression

**Figure 3:**
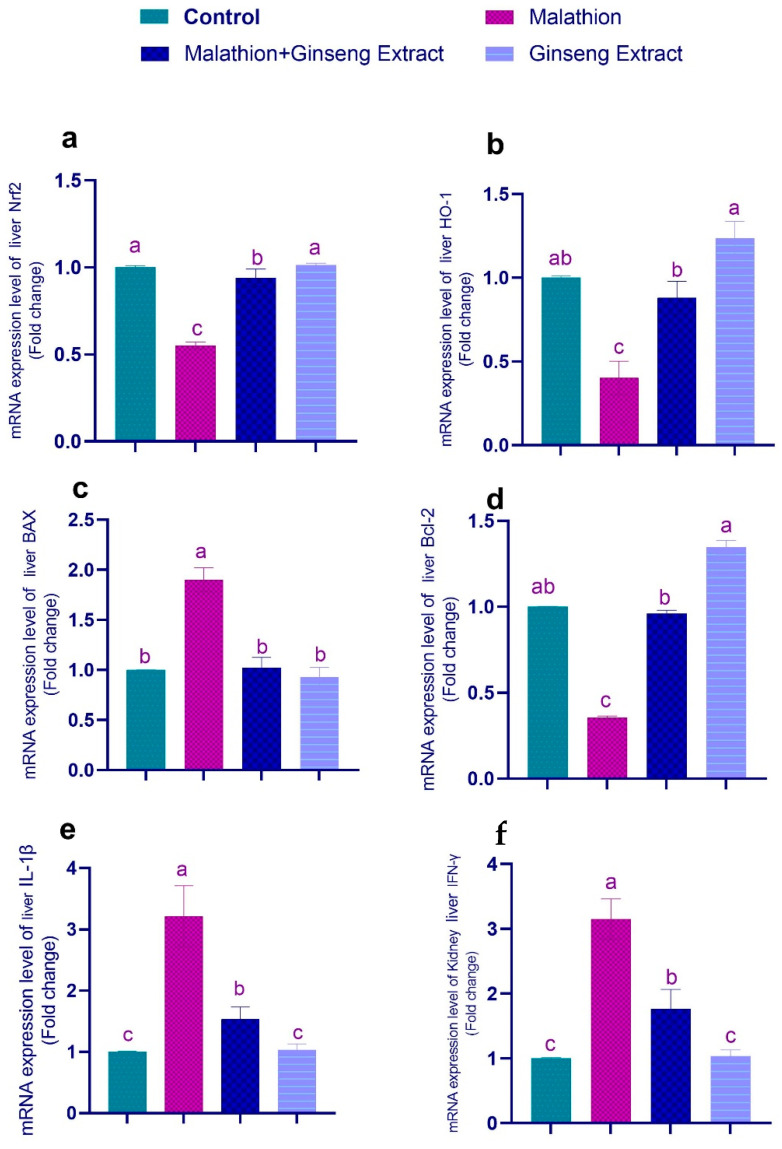
Ginseng^®^’s effect on hepatic inflammatory markers caused by malathion. Nrf2 (**a**), HO-1 (**b**), Bax (**c**), Bcl-2 (**d**), IL-1β (**e**), IFN-γ (**f**). Data were expressed as mean ± SEM. ^a–c^. Mean values within the columns having different superscript letters are significantly different at *p* < 0.05. *n* = 7 rats.

**Figure 4:**
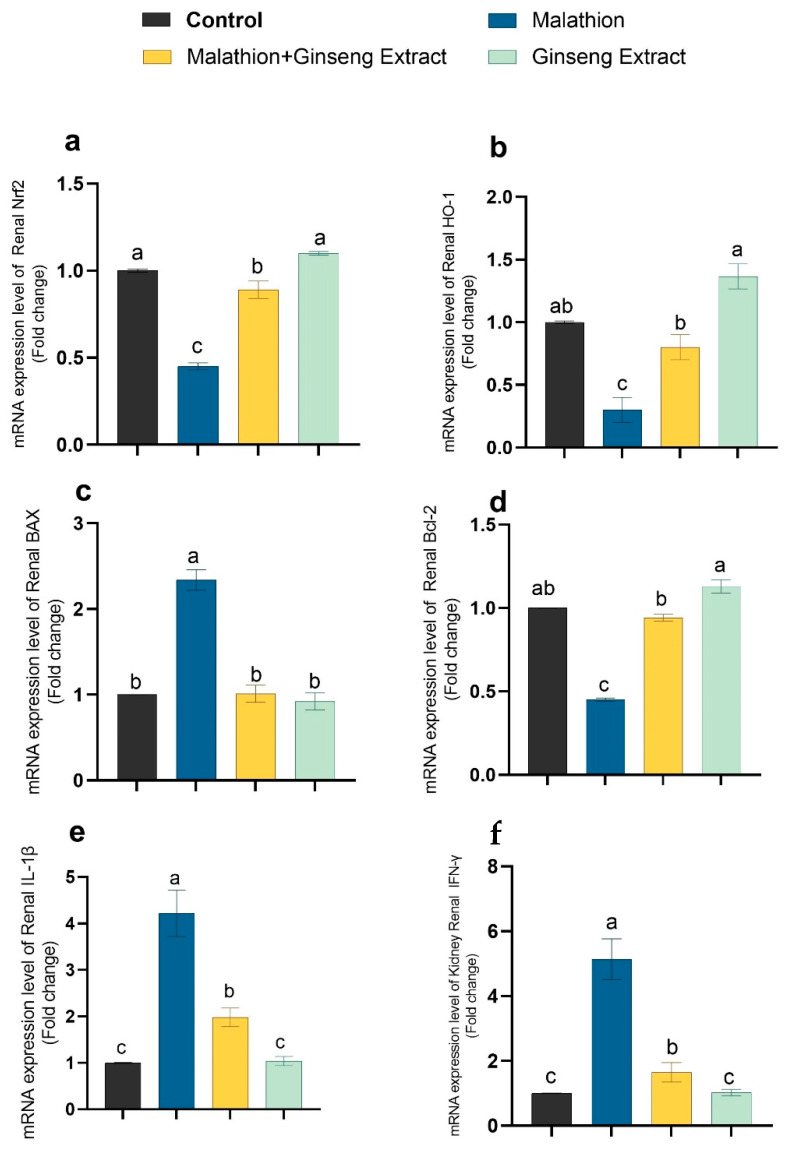
Ginseng^®^’s effect on the renal inflammatory markers caused by malathion. Nrf2 (**a**), HO-1 (**b**), Bax (**c**), Bcl-2 (**d**), IL-1β (**e**), IFN-γ (**f**). Data were expressed as mean ± SEM. ^a–c^. Mean values within the columns having different superscript letters are significantly different at *p* < 0.05. *n* = 7 rats.

Figure 3 and Figure 4 show that malathion-treated rats’ livers and kidneys had significantly higher IL-1, Bax, and IFN-mRNA expression than other control groups, and Nrf2, Bcl-2, and HO-1 were downregulated in malathion-treated groups. The co-treatment of Ginseng^®^ and malathion resulted in significant restoration of deviated genetic expression to control levels.

### 3.5. 8-OHdG and TNF-Alpha 

**Figure 5:**
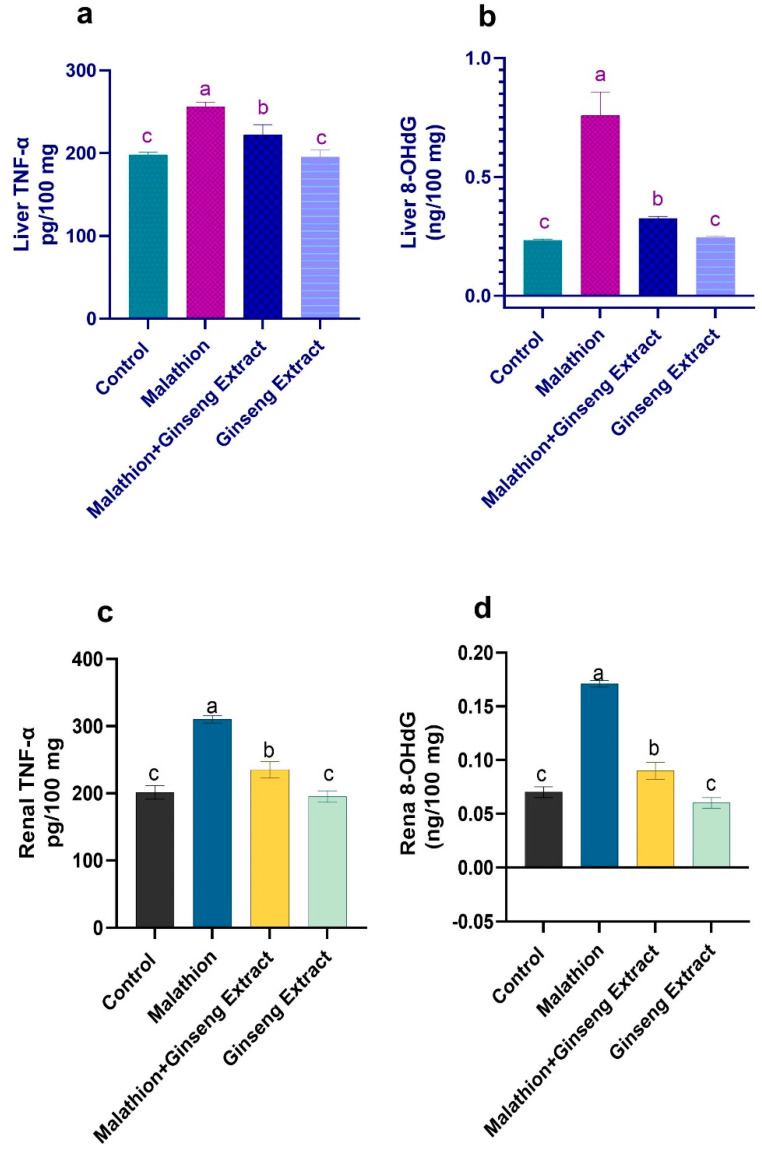
Ginseng^®^’s effect on oxidative stress markers caused by malathion in hepatic and renal tissue (**a**) Liver TNF-α (**b**) liver 8-OHdG (**c**) Kidney TNF-α (**d**) Kidney 8-OHdG. Data were presented as mean ± SEM. ^a–c^. Mean values within the columns having different superscript letters are significantly different at *p* < 0.05. *n* = 7 rats.

TNF-α and 8-OHdG were increased in the liver and kidneys by malathion administration, indicating a general inflammatory state. The 8-OHdG and TNF- levels were significantly restored when Ginseng^®^ and malathion were taken together, as shown in Figure 5.

### 3.6. Histopathological Findings

**Figure 6:**
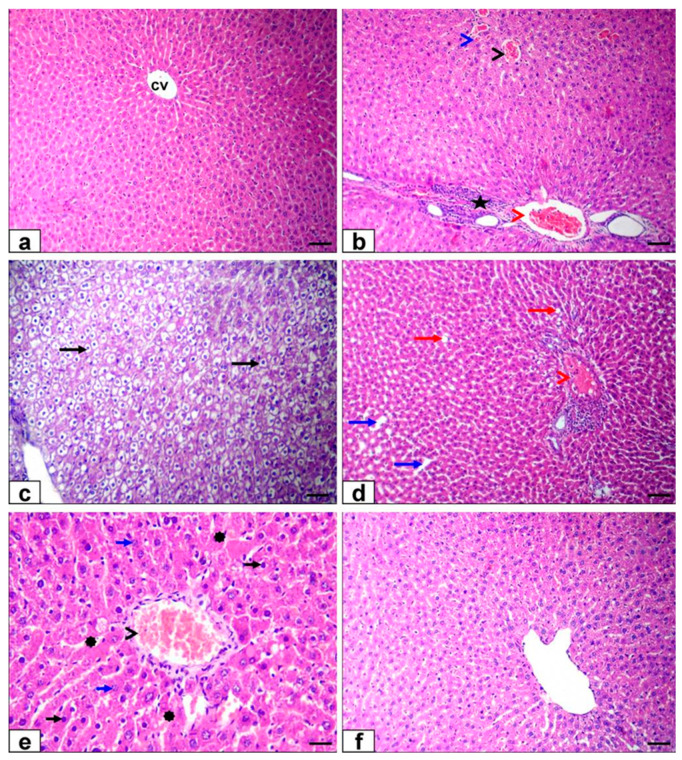
Photomicrograph of HE-stained liver slices: (**a**) liver of control group showing normal tissue structure cv (Central vein).(**b**–**e**) Malathion-treated rats’ livers exhibiting congestion of venous side of the central circulation vein (black arrowhead), portal vein (red arrowhead), and hepatic sinusoid (blue arrow), and infiltration of inflammatory cells in the portal area (star) beside diffuse hepatocytes deterioration of (black arrows), mild sharp edge outline vacuoles (red arrows), and mild hepatic sinusoids dilatation (blue arrows). In addition, necrobiotic changes of hepatocytes (pyknotic nucleus (short black arrows), lysis of nuclear chromatin (short blue arrows), and disappearance of a nucleus with an increase in cytoplasmic eosinophilia of some hepatocytes (asterisk) (**f**) The histological structure of rats treated with Ginseng^®^ and malathion was practically normal. Bar of (**a**–**d**,**f**) =100 µm and (**e**) =50 µm.

The liver of the control and Ginseng^®^ treated rats showed the standard histological structure of hepatic lobules and central vein (Figure 6a). The liver sections of malathion-treated rats exhibited severe congestion of the hepatic venous side of the circulation (central vein, portal vein, and hepatic sinusoid) and moderate mononuclear inflammatory cell infiltration in the portal area (Figure 6b) beside diffuse hydropic degeneration of hepatocytes (Figure 6c), mild sharp edge outline vacuoles and mild dilatation of hepatic sinusoids (Figure 6d). Moreover, there were necrobiotic changes in hepatocytes; some hepatocytes contained pyknotic nuclei, which were small, round, and coarsening of the heterochromatin. Others were lysis of nuclear chromatin and disappearance of nucleus forming ghost nuclei with an increase in cytoplasmic eosinophilia of some hepatocytes (Figure 6e). 

**Figure 7:**
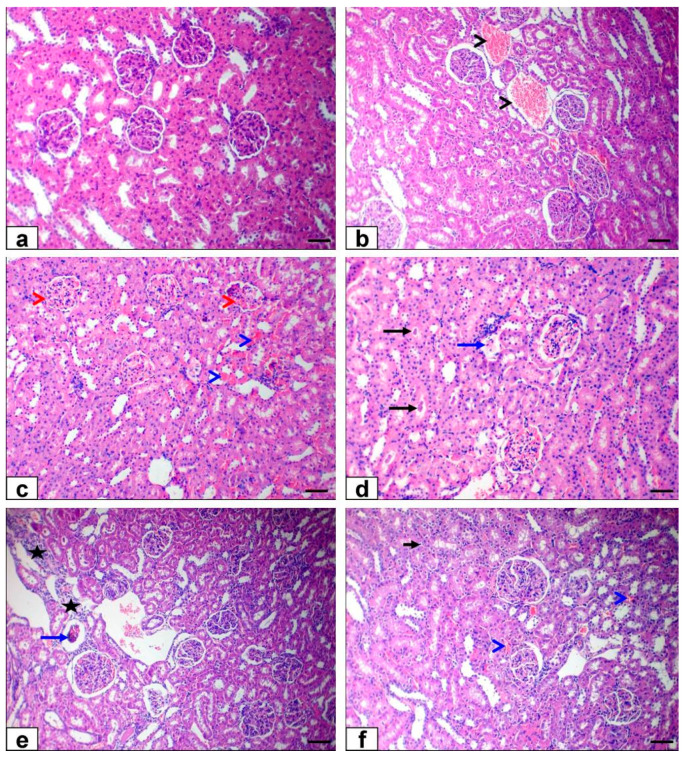
Photomicrograph of HE-stained kidney slices: (**a**) kidney of control group showing normal tissue structure (**b**–**e**). Malathion-treated rats’ kidneys displaying congestion of renal blood vessels (black arrowheads), inter-tubular capillaries (blue arrowheads), and glomerular capillaries (red arrowheads) beside necrotic glomerulus (blue arrow), hyaline casts (black arrows) in the lumen of renal tubules and interstitial mononuclear cell infiltrations (stars) (**f**). Ginseng^®^-treated rats with malathion showed nearly normal histological structure with mild hydropic epithelial cell degenerations (short black arrow) and mild congestion of inter-tubular capillaries (blue arrowheads). Bar = 100 µm.

The Ginseng^®^ with malathion-treated group showed nearly normal histological structure (Figure 6e). The kidney sections of the control and Ginseng^®^ treated rats displayed normal structure of the cortex and medulla (Figure 7a). The kidney sections of malathion-treated rats exhibited severe congestion of renal blood vessels (Figure 7b), inter-tubular capillaries, and glomerular capillaries (Figure 7c) beside necrotic glomerulus, hyaline casts in the lumen of renal tubules (Figure 7d), and mild interstitial mononuclear cell infiltrations (Figure 7e). The co-treatment of Ginseng^®^ with malathion exhibited a nearly normal tissue architecture with minor hydropic renal epithelial cell degeneration and mild congestion of inter-tubular capillaries (Figure 7f).

## 4. Discussion

Malathion is a widespread organophosphate insecticide used to control various insects [42]. Malathion and its metabolites can induce oxidative stress and impairment of hepatic and renal function [43], destroying the cellular membranes, causing DNA damage, and increasing ROS production, leading to biological system oxidative damage [44,45]. The gastrointestinal contents and adipose tissue both had high levels of malathion. Biliary excretion appears to be a primary route of elimination for metabolites, as MCA was identified at a relatively high level. The kidneys had the highest quantities of metabolites for DCA and MCA [46]

Many antioxidants are used against malathion toxicity, and the purpose of this study was to see if Ginseng^®^ could protect against malathion poisoning. The liver enzymes play a critical role in regulating physiological processes, for example, biosynthesis of macromolecules, cellular metabolism, and detoxification [47]. In our study, the malathion administration caused significant upsurges in the serum ALT, AST, ALP, LDH, and ACP activities, and AchE and paraoxonase activity compared to a control group. This may be due to the ability of malathion to induce oxidative stress and production of ROS, causing liver damage and necrosis, leading to the liberation of these enzymes from hepatic cells to blood [48,49]. For mediating the bio-activation of thiono-organophosphates, the liver is the most active metabolizing organ [50]. It is one of the essential malathion poisoning targets [51]. Blasiak et al. [52] have reported that malathion has an initial cytotoxic action in human lymphocytes, causing cellular death without causing DNA damage. Still, its active metabolites, malaoxon and isomalathion, act on DNA, breaking its chains.

These results follow [53,54,55], who reported that malathion administration induced liver enzyme activities. Inflammatory cytokines, metabolic dysfunction, apoptosis, and gene expression modulation are all factors that contribute to malathion’s hepato-renal toxicity [3]. In agreement with [1,54], our results also showed significant decreases in the serum TP and albumin. This may be explained by the ability of malathion to induce liver damage and decrease the synthesis, digestion, and absorption of protein [56] because the liver is the main site for plasma protein synthesis. Additionally, our results exhibited a considerable rise in cholesterol and a reduction in TG levels. This may be due to the ability of pesticides to block the bile duct and decrease cholesterol secretion in the intestine [57] or to the inhibition of the pancreatic function by malathion leading to poor absorption of lipids [58]. These findings are in line with [54], who reported a substantial rise in cholesterol and a drop in TG in male mice injected with malathion for six days. In renal function tests, our study revealed significant increments in serum urea, creatinine, and uric acid levels in malathion intoxicated rats. These increments indicate renal dysfunction [59]. This may be due to the induced renal oxidative damage with increased ROS production and decreased antioxidants [60]. It may also be due to glomerular filtration deficiency, whereby excretion decreases and serum levels rise [61]. In addition, many medicines can change uric acid levels, affecting uric acid net reabsorption in the proximal tubules [62]. These results agree with the results of [1,63,64]. 

The hepatorenal induced malathion toxicity is related to the induction of oxidative stress [43], so our previously reported biochemical changes can be proven by our observations that revealed significant reductions in hepatorenal GSH level and antioxidant enzymes activities (GPX, SOD, and CAT) associated with noteworthy increments in MDA and NO levels in the malathion intoxicated group. In addition, when malathion is converted to malaoxon, it is known to produce a lot of reactive oxygen species in the liver [65].

These results are consistent with [66,67,68] in hepatic tissue, and [1,69] in hepatic and renal tissues. Our findings have verified other studies that corroborate that the organophosphorus administration causes a disturbance in hepatic and renal tissues [70]. Organophosphorus exposure is connected with many health problems, including oxidative stress and ROS overproduction [49]. 

Malathion’s toxicity is exacerbated by its metabolites and pollutants. Malathion’s principal source of toxicity is malaoxon, which is formed by the oxidation of malathion in mammals, animals, and plants and is 40 times more acutely hazardous than malathion [71,72]. Malathion is transformed to malaoxon via oxidative sulfuration, which is mediated by a microsomal system of enzymes known as mixed-function oxidases (MFO), one of which is cytochrome P450 (CYP450) [65]. The liver has a robust oxidative metabolism and a lot of CYP450 activity, essential for xenobiotic biotransformation [73]. High levels of ROS are produced during the biotransformation processes of malathion into malaoxon. Malathion is also detoxified through glutathione conjugation reactions. Malathion exposure was found to reduce GSH levels, raise GSSG levels, and lower the GSH/GSSG ratio [74].

The malathion-induced oxidative stress can also increase the nitric oxide synthase enzyme activity in the rat liver and nitric oxide production [14]. Furthermore, as reported in this study, the malathion-induced hepatorenal histopathological changes support our biochemical results and the oxidative changes. The liver showed congestion of blood vessels, hepatic degeneration, and necrosis associated with inflammatory cell infiltration results [54,75]. Additionally, the renal tissue showed severe congestion of renal blood vessels and glomerular necrosis with the presence of hyaline casts in a tubular lumen in agreement with [54]. Furthermore, an initial effect of malathion’s toxic compounds is that it activates Kupffer cells in the liver, resulting in enhanced MPO activity and the release of pro-inflammatory cytokines such as IL-1β, IL-6, and INF-γ. For the reason inflammatory responses are implicated in liver injury via inducing hepatosteatosis, the function of inflammatory reactions in toxicological processes is of great interest [76].

Our results showed that co-treatment of Ginseng^®^ to malathion intoxicated rats ameliorated the hepatorenal toxicity induced by malathion. The Ginseng^®^ biochemical hepatoprotective related outcomes were proved by significant reductions in the serum ALT, AST, ALP, LDH, ACP, AchE, and paraoxonase activities, cholesterol levels with substantial increases in TP, albumin, and TG levels when compared with the malathion intoxicated group. Additionally, the Ginseng^®^ treatment alone presented a remarkable upsurge in TP levels compared with the control. Our parallel findings show that Ginseng^®^ can protect the liver against CCL4 toxicity [77], D-galactosamine/lipopolysaccharide [78], Fipronil [79], and cyhalothrin [80], and that Ginsenoside Rg5 improves AChE in the brain cortex [81,82].

Although the liver is the primary source of paraoxonase, it has also been found in the kidney, heart, and brain [83]. After being synthesized in the liver, the paraoxonase enzyme, an antioxidant that prevents LDL oxidation, is transported along with HDL in the plasma [84]. Paraoxonase activity was improved by Ginseng^®^ supplementation [85], which supports our Paraoxonase findings. Thus, the liver is the primary metabolizing site for thiono-organophosphate biotransformation, with the kidney contributing to hazardous product removal [22,23]. Many illnesses linked to organophosphorus exposure are preceded by excessive production of reactive oxygen species (ROS) and oxidative stress [49,86].

Furthermore, the renal biochemical parameters showed that co-administration of Ginseng^®^ led to substantial decrements in serum urea, creatinine, and uric acid levels compared with malathion only. These findings accord with the findings that indicate the Ginseng^®^ renoprotective effect against gentamicin sulfate [77] and Cisplatin [79,87,88]. The antioxidant properties of Ginseng^®^ have long been recognized due to its ability to increase the expression of antioxidant enzyme genes that scavenge reactive oxygen species. Ginseng^®^ increases antioxidant enzyme activity and free radical scavenging [89]. SOD and GPx, as well as heme oxygenase-1 (HO-1), were discovered to be boosted by Ginseng^®^’s ability to increase the activity of self-antioxidant enzymes such as SOD and GPx [90,91], in addition to lipid peroxidation inhibition [92,93]. Ginseng^®^ recovers the glomerular filtration, leading to the increased thickness of the basement membrane glomeruli, as reported by [77]. Ginseng^®^’s hepato-renal protective impact can be traced back to its biochemical and pharmacological capabilities, which include anti-inflammatory and anti-hyperlipidemic properties [94], and antioxidant effects as free radical scavenging and stimulating the activities of antioxidant enzymes, which play a role in scavenging of ROS as reported in our study, and in [89]. Additionally, these biochemical results may be confirmed by the reported antioxidant effects of co-treatment with Ginseng^®^ in our study, showing significant increases in GSH quantities and antioxidant enzyme activities (GPX SOD and CAT) associated with marked reductions in MDA and NO levels in comparison with the malathion intoxicated group. These results agree with the results of [95] in renal tissue and [79,80] in hepatic and renal tissue.

The hepato-renal protective effect of Ginseng^®^ against malathion exhibited histopathology as Ginseng^®^ improved and ameliorated the toxic alterations caused by malathion to nearly normal appeared organs. These results are supported by other studies on the protective effect of Ginseng^®^ against hepatorenal damage induced by fipronil as reported by [79] and hepatic injury caused by cyhalothrin as reported by [80].

TNF-α is a cytokine that promotes inflammation elaborated in innate and acquired immunity, cell proliferation, tissue necrosis, and apoptosis [96]. In mutagenesis damage, 8-OHdG is the most prevalent base modification and is a biomarker for DNA oxidative stress [97]. In this study, the malathion intoxication increased the serum levels ofTNFα significantly following [68] due to enlargement of sinusoids, mononuclear cell infiltration, and hepatic necrosis caused by malathion as reported by [1]. Additionally, it showed a significant increase in 8-OHdG levels in agreement with [98] compared with the control rats. This increment in serum 8-OHdG level approved the role of malathion in exhibiting DNA damage due to induction of genotoxicity and chromosomal aberrations [99].

On the other hand, the co-administration of Ginseng^®^ to malathion intoxicated rats led to a significant decrease in TNFα in the same harmony with [87,100] against N-acetyl-p-aminophenol-induced hepatotoxicity and cisplatin-induced renal toxicity, respectively. Furthermore, the increment in 8-OHdG levels is parallel with [101] against cyclosporine nephrotoxicity. The ameliorating effect of Ginseng^®^ can be returned to its antioxidant properties as previously mentioned due to it containing vital constituents such as ginsenosides, polyacetylenes, flavonoids, and phenolics [95] which are responsible for the protective effects of Ginseng^®^ against various diseases [102]. 

Malathion’s acute toxicity affects the neurological system due to the inactivation of AChE and BChE enzymes. It could be concluded that Ginseng^®^ has antioxidant effects against malathion toxicity by improving hepatic, renal biochemical parameters, and oxidative biomarkers which decrease the TNFα and serum 8-OHdG levels. [1]. 

We discovered significant upsurges in the expression of IL-1β, IFN-γ, and Bax expression. Despite this, mRNA expression of the Bcl-2, Nrf2, and HO-1 genes was decreased, indicating apoptosis and inflammatory response. Furthermore, these investigations establish a connection between malathion-induced inflammatory reactions in the liver and insulin resistance [103]. Ginseng^®^’s antioxidant activities and subsequent ROS scavenging, suppression of NF-B activation, and cytokine release are responsible for the enhanced apoptotic rate in cotreated groups. To protect against oxidative stress-induced cell death in neuroblastoma cells, Ginseng^®^ P53 and caspase-3 were downregulated, whereas the anti-apoptotic Bcl2 increased [104].

In histopathological examination, Ginseng^®^ supplementation reduced most pathological microscopic changes related to malathion exposure, which supports the previously discussed Ginseng^®^ protection mechanisms against hepato-renal damage. Our result was supported by [105], who showed malathion’s adverse effects on the kidney and hepatic tissue. In the same way, the co-treatment with Ginseng^®^ reinstates typical hepatic architecture, as stated by [80].

## 5. Conclusions

Ginseng^®^, as a therapeutic solution including multiple active components, was found to be effective in protecting against the biochemical, oxidative, and inflammatory effects of malathion. This is probably via restoring metabolic parameters, increasing antioxidant defense systems, and lowering inflammatory mediator production.

## Figures and Tables

**Table 1:** The effects of malathion and Ginseng^®^ either alone or in combination on growth performance.

	Control	Malathion	Malathion + Ginseng^®^	Ginseng^®^
Initial body Weight (g)	145.02 ± 4.1	143.15 ± 3.4	147.1 ± 3.4	145.2 ± 3.5
Final body Weight (g)	265.14 ± 3.15 ^a^	231.2 ± 4.9 ^c^	245.65 ± 5.2 ^b^	266.15 ± 4.1 ^a^
Bodyweight gain	120.12 ± 3.8 ^a^	88.08 ± 3.1 ^c^	98.55 ± 2.15 ^b^	120.95 ± 3.9 ^a^
Absolute Weight of Liver (g)	4.59 ± 0.12	4.41 ± 0.10	4.83 ± 0.6	5.01 ± 0.1
Relative liver weight (g/100 g BW)	1.73 ± 0.10	1.90 ± 0.11	1.96 ± 0.12	1.88 ± 0.11
Absolute Weight of Kidney (g)	1.19 ± 0.11	1.22 ± 0.012	1.25 ± 0.01	1.26 ± 0.01
Relative Weight of Kidney (g/100 g BW)	0.44 ± 0.01	0.52 ± 0.01	0.50 ± 0.01	0.47 ± 0.1

Data were expressed as mean ± SEM. ^a–c^ Means within the same row carrying different superscript letters are significantly different at *p* < 0.05. *n* = 7 rats.

**Table 2:** The effects of malathion and Ginseng^®^ either alone or in combination on the activities of serum liver enzymes and other biochemical parameters in male rats.

	Control	Malathion	Malathion + Ginseng^®^	Ginseng^®^
AST (U/mL)	81.39 ± 3.2 ^c^	181.50 ± 10.1 ^a^	97.01 ± 5.4 ^b^	77.29 ± 3.2 ^c^
ALT (U/mL)	35.50 ± 1.2 ^c^	80.92 ± 3.2 ^a^	52.30 ± 3.1 ^b^	34.92 ± 1.4 ^c^
ALP (U/L)	86.05 ± 3.1 ^c^	212.61 ± 6.45 ^a^	107.81 ± 5.4 ^b^	85.67 ± 3.14 ^c^
LDH (U/L)	205.6 ± 10.14 ^c^	453.54 ± 10.4 ^a^	321.88 ± 12.4 ^b^	201.16 ± 11.2 ^c^
ACP(U/L)	105.12 ± 10.2 ^c^	172.3 ± 12.14 ^a^	129.34 ± 9.45 ^b^	101.14 ± 5.9 ^c^
TP (g/L)	5.15 ± 0.15 ^a^	3.59 ± 0.21 ^c^	4.54 ± 0.12 ^b^	5.23 ± 0.5 ^a^
Albumin (g/L)	4.06 ± 0.4 ^a^	2.67 ± 0.09 ^c^	3.74 ± 0.14 ^b^	4.19 ± 0.45 ^a^
Cholesterol (mg/dL)	80.23 ± 5.45 ^c^	156.56 ± 7.1 ^a^	112.54 ± 6.14 ^b^	78.31 ± 3.4 ^c^
TG (g/L)	111.39 ± 10.2 ^a^	71.16 ± 4.1 ^c^	90.97 ± 5.14 ^b^	105.66 ± 7.4 ^a^
Urea (mg/dL)	22.05 ± 1.4 ^c^	77.69 ± 2.4 ^a^	43.48 ± 1.5 ^b^	20.19 ± 1.5 ^c^
Creatinine (mg %)	0.66 ± 0.01 ^c^	2.15 ± 0.1 ^a^	1.90 ± 0.04 ^b^	0.52 ± 0.01 ^c^
Uric acid (mg/dL)	25.71 ± 1.2 ^c^	84.06 ± 5.1 ^a^	44.34 ± 2.1 ^b^	23.07 ± 2.1 ^c^
Paraoxonase (U/L)	171.12 ± 12.5 ^a^	122.45 ± 12.15 ^c^	139.9 ± 5.15 ^b^	175.14 ± 10.2 ^a^
AChE (U/L)	240.15 ± 12.15 ^a^	86.15 ± 4.5 ^c^	133.15 ± 7.15 ^b^	238.15 ± 8.45 ^a^
Ammonia (μmol/L)	133.25 ± 5.45 ^c^	263.15 ± 15.2 ^a^	189.45 ± 10.2 ^b^	132.25 ± 10.25 ^c^

AST: aspartate aminotransferase, ALT: alanine aminotransferase, ALP: alkaline phosphatase, LDH: lactate. Dehydrogenase, ACP: acid phosphatase, TP: total protein, and TG: triglycerides. AChE: Acetylcholinesterase. Data expressed mean ± SEM. ^a–c^ Means within the same row carrying different superscript letters are significantly different at *p* < 0.05. *n* = 7 rats.

## Data Availability

Upon request from the corresponding author.

## Supplementary Materials

The following supporting information can be downloaded at: https://www.mdpi.com/article/10.3390/life12050771/s1, Table S1: Primers for gene expression by RT-PCR.
