# Supplementary material for: Ginseng® Alleviates Malathion-Induced Hepatorenal Injury through Modulation of the Biochemical, Antioxidant, Anti-Apoptotic, and Anti-Inflammatory Markers in Male Rats
Source: Life (Basel). 2022 May 23;12(5):771. doi: 10.3390/life12050771 (PMC9144712; doi:10.3390/life12050771)
Supplement: Supplementary file 1 [file life-12-00771-s001.zip › life-1708364-supplementary.pdf]

Table S1. Primers for gene expression by RT-PCR

**Gene**	**Direction**	**Primer sequence**	**Accession number**
Bax	Sense	GGCGAATTGGCGATGAACTG	NM_017059.2
	Antisense	ATGGTTCTGATCAGCTCGGG	
Bcl-2	Sense	GATTGTGGCCTTCTTTGAGT	NM_016993.1
	Antisense	ATAGTTCCACAAAGGCATCC	
GAPDH	Sense	TCAAGAAGGTGGTGAAGCAG	NM_017008.4
	Antisense	AGGTGGAAGAATGGGAGTTG	
IL-1β	Sense	ACC CAA GCA CCT TCT TTT CCT T	NM_031512.2
	Antisense	ACG GGA AAC CCA TCA CCA T	
HMOX1	Sense	AGCATGTCCCAGGATTTGTC	NM_012580.2
	Antisense	TCACCAGCTTAAAGCCTTCC	
NRF2	Sense	TTGTAGATGACCATGAGTCGC	NM_031789
	Antisense	TGTCCTGCTGTATGCTGCTT	
IFN-γ	Sense	AGGTGAACAACCCACAGAT	NM_138880.3
	Antisense	CTTCTTATTGGCACACTCTCTAC	

Bax, Bcl-2-associated X protein. *Bcl-2*, B-cell lymphoma 2. GAPDH, glyceraldehyde-3-phosphate dehydrogenase. Nuclear factor, erythroid derived 2, like 2 (Nrf2) 1. IFN-γ, Interferon-gamma. *HMOX1*, Haemoxygenase-1.
